# Probable Cold Agglutinin Disease Presenting With Profound Anemia and Cardiopulmonary Decompensation in a 93-Year-Old Woman: A Case Report

**DOI:** 10.7759/cureus.114136

**Published:** 2026-08-07

**Authors:** Martyna Dziurzyńska, Patryk Dziurzyński, Hubert Piwar, Monika Lis, Magdalena Puziuk

**Affiliations:** 1 Department of Internal Medicine and Gastroenterology, Masovian Bródnowski Hospital, Warsaw, POL; 2 Department of Gastroenterology, Bielanski Hospital, Warsaw, POL; 3 Department of Medicine, Dr. Anna Gostynska Wolski Hospital, Warsaw, POL; 4 Department of Medicine, Masovian Bródnowski Hospital, Warsaw, POL; 5 Department of Medicine, Dr. Aleksander Majkowski District Hospital, Kartuzy, POL

**Keywords:** autoimmune hemolytic anemia, cold agglutinin disease, complement-mediated hemolysis, immunoglobulin m, monoclonal protein, older adult, severe anemia

## Abstract

Cold agglutinin disease (CAD) is an uncommon, complement-mediated autoimmune hemolytic anemia that predominantly affects older adults. We report a 93-year-old woman with ischemic heart disease, persistent atrial fibrillation treated with rivaroxaban, major valvular disease, and limited mobility who presented with progressive leg edema, dyspnea, chest pain, fatigue, and profound normocytic anemia. Her hemoglobin was 5.1 g/dL, compared with 9.7 g/dL approximately four months earlier. The initial evaluation focused on possible occult bleeding and cardiac decompensation. By the second day of the acute-care episode, the finalized blood-group confirmation result returned from the regional blood center reportedly noted cold-reactive antibodies, providing an early clue to cold-antibody-mediated hemolysis and redirecting the diagnostic work-up. No overt bleeding was identified, although gastrointestinal assessment remained incomplete. Subsequent hemolysis testing showed a lactate dehydrogenase level of 401 U/L, reticulocytes of 4.78%, and a positive direct antiglobulin test with C3d reactivity. Serum immunofixation identified an immunoglobulin M kappa monoclonal protein. Directed questioning then elicited long-standing cold intolerance, severe pain after cold food or beverages, and cold-triggered limb pain. Because the cold agglutinin titer and thermal amplitude were unavailable, the findings were considered strongly supportive of probable CAD associated with an immunoglobulin M-related clonal process rather than proof of a fully characterized hematologic disorder. Evaluation for an underlying cause was limited by the absence of on-site hematology services, and lymphoma or another marrow-based neoplasm could not be excluded. One packed red-cell unit was completed in the emergency department; a second was stopped when dyspnea developed against a background of marked edema and cardiac disease, and two further units were later given under diuretic cover. With transfusion support, diuresis, cold avoidance, and other supportive care, hemoglobin increased to 9.3 g/dL and cardiopulmonary symptoms improved. By discharge, she was alert and oriented to self and her surroundings and could ambulate with assistance or a walker. At approximately 12 weeks after discharge, she remained clinically stable. After discussion of the expected benefits and burdens, the patient, supported by her family, chose not to pursue invasive hematologic evaluation because its results were unlikely to alter the planned conservative management. This case emphasizes directed inquiry about temperature-related symptoms and careful calibration of diagnostic certainty when formal CAD testing is incomplete.

## Introduction

Cold agglutinin disease (CAD) is an uncommon complement-mediated autoimmune hemolytic anemia that occurs mainly in older adults. Clinical manifestations include fatigue, dyspnea and other consequences of anemia, as well as cold-induced circulatory symptoms such as acrocyanosis or painful extremities. A Norwegian population-based study estimated an incidence of approximately one case per million persons per year, and institutional cohorts show substantial burdens of severe anemia and red-cell transfusion [1,2].

Pathogenic cold agglutinins are usually immunoglobulin M (IgM) antibodies. They bind erythrocytes in cooler parts of the circulation, cause agglutination, and activate the classical complement pathway. Complement fragments remain on the erythrocytes after rewarming, producing a characteristically C3d-reactive direct antiglobulin test (DAT) and predominantly hepatic erythrocyte clearance [2,3]. Thermal amplitude is the highest temperature at which the antibody remains reactive. It helps determine whether ordinary peripheral cooling can trigger clinically important disease [2].

CAD is now understood as an IgM-related clonal B-cell disorder rather than a purely idiopathic autoimmune phenomenon. Monoclonal IgM, most often kappa restricted, is common and prompts evaluation of the underlying clone, although it does not by itself identify a specific lymphoproliferative neoplasm [1,2,4]. Diagnosis can be difficult in older patients because cardiac disease, anticoagulant use, and possible blood loss provide competing explanations for anemia and dyspnea. We present a 93-year-old woman in whom probable CAD was initially obscured by these alternatives and the decisive cold-related history emerged only after targeted questioning.

## Case presentation

A 93-year-old woman was referred from primary care because of more than one week of progressive bilateral leg edema with serous leakage, severe fatigue, dyspnea, and chest pain during dressing and standing. Symptoms improved with rest and sublingual nitrate. At presentation, she appeared mildly confused; hearing impairment and mild memory impairment were also noted, and the history was supplemented by her family. Her history included ischemic heart disease, an inferior-wall myocardial infarction treated with percutaneous coronary intervention and a drug-eluting stent to the right coronary artery in 2017, persistent atrial fibrillation treated with rivaroxaban, dyslipidemia, chronic degenerative disease, glaucoma, cataract, and mobility limitation requiring a walker. The available medication record also documented chronic diuretic, lipid-lowering, analgesic, electrolyte-replacement, and topical glaucoma therapy, but exact doses were unavailable. The combined patient-family history included intermittent mild epistaxis but no reported gastrointestinal bleeding, change in bowel habits, vomiting, dysphagia, abdominal pain, or syncope. Family history was not documented. Approximately four months earlier, during hospitalization after head trauma, her hemoglobin had been 9.7 g/dL. The major clinical events are summarized in Table 1.

**Table 1:** Clinical timeline DAT: direct antiglobulin test; LDH: lactate dehydrogenase; TIBC: total iron-binding capacity; UIBC: unsaturated iron-binding capacity

Relative time	Clinical event
Approximately four months before Day 0	Hospitalization after head trauma; hemoglobin 9.7 g/dL. No hemolysis studies from that episode were available.
Months before Day 0	Cold intolerance, an unusually warm home environment, pain after cold food or beverages, and cold-triggered limb pain were present but were elicited only later by directed questioning.
Day 0: emergency presentation	Progressive edema, fatigue, dyspnea, and exertional chest pain; hemoglobin 5.1 g/dL. She appeared mildly confused, and the initial history was supplemented by her family. Imaging showed cardiomegaly, small pleural effusions, and no overt pulmonary venous congestion.
Day 0-1	Against a background of marked peripheral edema and structural heart disease, one packed red-cell unit was completed. Dyspnea developed during a second unit, which was stopped; a control chest radiograph showed increased pulmonary congestion and pleural fluid.
By Day 2 of the acute-care episode	The finalized blood-group confirmation result returned from the regional blood center reportedly noted cold-reactive antibodies, providing an early serologic clue and prompting a hemolysis-directed evaluation.
Days 2-14	Locally available investigations showed LDH 401 U/L, reticulocytes 4.78%, DAT reactivity with C3d and IgM, serum cold-reactive antibodies, and an IgM-kappa monoclonal protein. Iron studies available from the admission showed serum iron 30 µg/dL, TIBC 341 µg/dL, UIBC 311 µg/dL, ferritin 41.7 ng/mL, and calculated transferrin saturation 8.8%. Two additional packed red-cell units were completed under diuretic cover; diuresis was intensified and ferric derisomaltose 1,500 mg was administered.
Day 14	Hemoglobin 9.3 g/dL with improvement in dyspnea, edema, mental status, and mobility.
Day 15	Discharged alert and oriented to self and her surroundings and able to ambulate with assistance or a walker; cold-avoidance advice and hematology referral were provided.
Approximately 12 weeks after discharge	Reported clinically stable under primary-care and family supervision. After discussion of expected benefits and burdens, the patient, supported by her family, chose conservative follow-up and declined invasive hematologic evaluation because its results were unlikely to change management.

At emergency presentation, hemoglobin was 5.1 g/dL, red-cell count 1.96 × 10⁶/µL, hematocrit 16.2%, and mean corpuscular volume 82.7 fL. Creatinine was reported as 1.28 mg/dL, urea as 125 mg/dL, and N-terminal pro-B-type natriuretic peptide as 1403.3 pg/mL. Initial upright chest radiography showed marked cardiomegaly, small bilateral pleural effusions, bibasal parenchymal opacities, and suspected nodules in the right perihilar and right lower lung fields; the formal report stated that pulmonary venous congestion was absent. Noncontrast abdominal computed tomography, obtained in the search for bleeding, could not determine a bleeding source and showed dilated hepatic veins and inferior vena cava, generalized cardiomegaly, pericardial and pleural fluid, and generalized subcutaneous edema.

**Figure 1:**
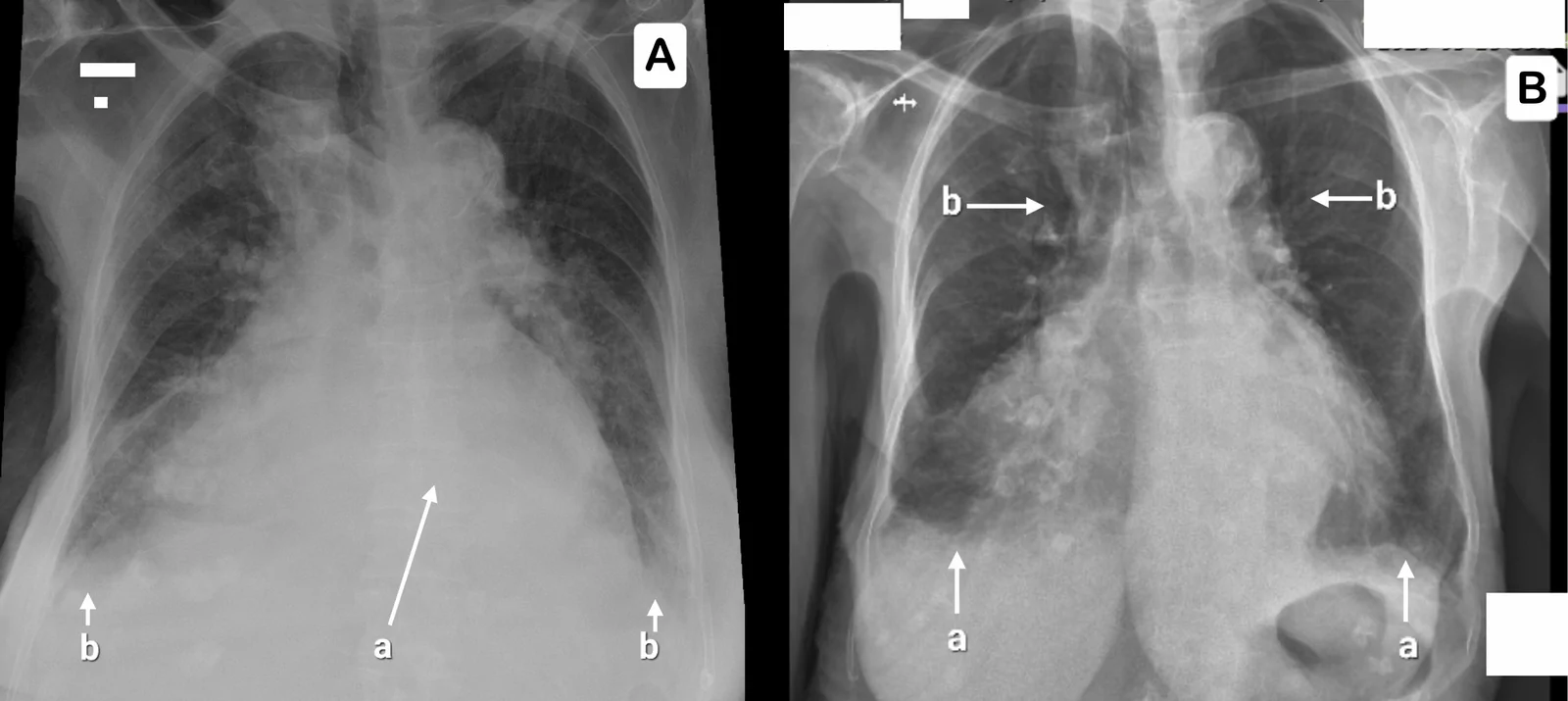
Chest radiographs before and during transfusion-associated dyspnea (A) Supine anteroposterior radiograph obtained on Day 1 after dyspnea developed during the second packed red-cell transfusion, showing marked cardiomegaly (arrow a), bilateral pleural effusions (arrows b), and pulmonary vascular congestion. (B) Upright posteroanterior radiograph obtained at emergency presentation on Day 0, showing cardiomegaly, small bilateral pleural effusions (arrows a), and perihilar opacities (arrows b); the formal radiology report stated that pulmonary venous congestion was absent. The panels are not directly comparable because they were obtained using different patient positions and acquisition techniques.

One packed red-cell unit was completed in the emergency department without an immediate complication. This occurred against a background of more than one week of worsening bilateral leg edema and major structural heart disease. During a second unit, dyspnea developed and the transfusion was stopped; the infused fraction and rate were not documented in the source summary. A control supine chest radiograph showed increased pulmonary vascular congestion and pleural effusions. On ward admission after the emergency transfusions, blood pressure was 136/81 mmHg, heart rate 83/min, and oxygen saturation 94% on room air. Dyspnea worsened during conversation and sitting. Examination found bibasal dullness and crackles, an empty rectal ampulla, edema to both knees, and marked painful hypersensitivity of the lower limbs. No formal transfusion-reaction investigation or immediate post-event hemolysis panel was documented. The paired radiographs document the findings at presentation and during transfusion-associated dyspnea (Figure 1).

Before the finalized blood-bank result became available, the investigation was centered on common sources of blood loss, particularly gastrointestinal bleeding in a patient receiving rivaroxaban. By the second day of the acute-care episode, the finalized blood-group confirmation result returned from the regional blood center reportedly noted cold-reactive antibodies. This was treated as an early clue rather than standalone proof of CAD. It redirected the evaluation toward immune hemolysis and its underlying cause.

**Figure 2:**
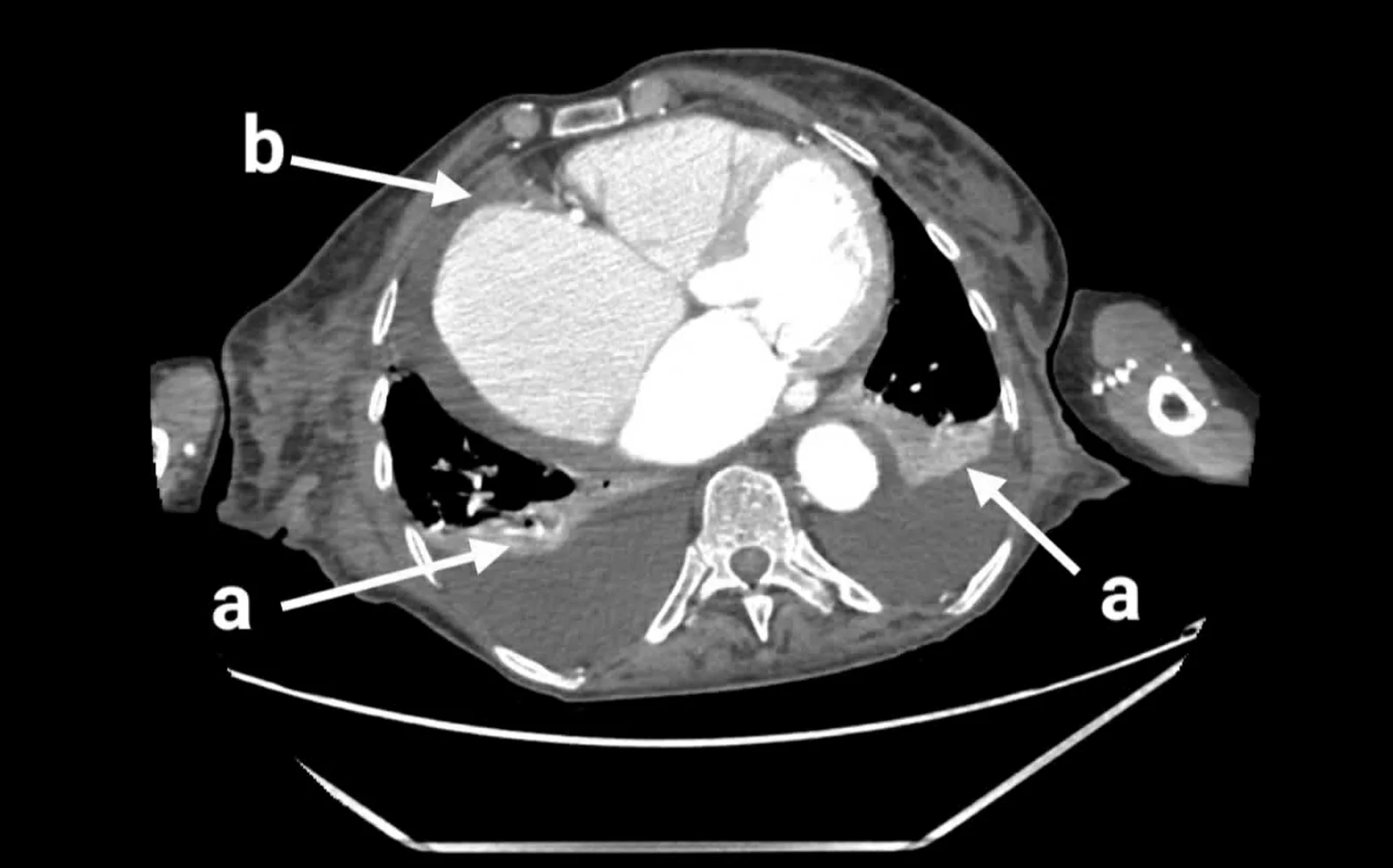
Contrast-enhanced chest computed tomography findings Axial contrast-enhanced chest computed tomography image obtained during admission showing bilateral pleural effusions with adjacent dependent atelectatic or consolidative change (arrows a), pericardial effusion along the anterior cardiac contour (arrow b), and marked enlargement of the cardiac chambers. These findings document the substantial cardiopulmonary fluid-overload context that provided a competing explanation for dyspnea; they are not diagnostic of CAD.

On the ward, two further packed red-cell units were completed under diuretic cover and diuresis was intensified. Iron studies available from the admission showed serum iron 30 µg/dL, total iron-binding capacity 341 µg/dL, unsaturated iron-binding capacity 311 µg/dL, and ferritin 41.7 ng/mL; the calculated transferrin saturation was 8.8%. This pattern supported concomitant iron deficiency with marked iron restriction, although absolute and inflammation-related functional components could not be separated. Ferric derisomaltose 1,500 mg was administered during the admission. The evaluation for occult gastrointestinal blood loss included the bleeding history, rectal examination, noncontrast abdominal computed tomography, and attempted endoscopy. Gastroscopy was started but stopped because of dyspnea and poor tolerance. Colonoscopy under anesthesia was not undertaken because of comorbidity and frailty, and the patient declined flexible sigmoidoscopy. No overt bleeding was identified, but occult gastrointestinal blood loss was not fully excluded. Contrast-enhanced computed tomography of the chest, abdomen, and pelvis showed findings consistent with biventricular heart failure, bilateral pleural effusions with dependent pulmonary changes, an enlarged nodular thyroid, hepatic cysts, and severe degenerative spinal disease. A representative CT image is shown in Figure 2.

Echocardiography demonstrated markedly enlarged atria, mild right ventricular enlargement with right ventricular free-wall hypokinesis and a tricuspid annular plane systolic excursion of 14 mm, a left ventricular ejection fraction of approximately 50%, moderate mitral regurgitation, and severe tricuspid regurgitation. The estimated tricuspid regurgitant gradient was 73 mmHg. A pericardial effusion up to 15 mm was hemodynamically insignificant. These findings documented substantial underlying structural heart disease; profound anemia was considered a likely contributor to, but not the sole established cause of, decompensation.

Further testing supported hemolysis: lactate dehydrogenase was 401 U/L, and reticulocytes were 4.78%. Total bilirubin was 1.10 mg/dL, direct bilirubin was 0.54 mg/dL, and calculated indirect bilirubin was 0.56 mg/dL; haptoglobin was unavailable. The available hospital summary documented a positive DAT with C3d and IgM reactivity and cold-reactive antibodies in serum. The original blood-bank report, reaction strength, monospecific IgG result, cold agglutinin titer, thermal amplitude, and specimen-handling details were unavailable. In conjunction with the chronic cold-triggered phenotype and subsequent IgM-kappa finding, the available results favored clinically relevant cold-antibody-mediated hemolysis over incidental cold reactivity, but they did not establish every formal criterion for CAD.

After the early blood-bank clue, the evaluation proceeded along two parallel tracks: unresolved occult blood loss and the cause of cold-antibody-mediated hemolysis. The etiologic differential included infection, autoimmune disease, and malignancy, particularly a B-cell lymphoproliferative disorder. Because the admitting hospital had no on-site hematology department, the work-up was limited to locally available investigations. Contrast-enhanced computed tomography of the chest, abdomen, and pelvis did not identify definite lymphadenopathy or hepatosplenomegaly. HIV antigen/antibody, hepatitis C antibody, and hepatitis B surface antigen tests were nonreactive. Serum immunofixation identified an IgM-kappa monoclonal protein; serum free kappa and lambda light chains were mildly increased, and serum protein electrophoresis showed low total protein and albumin with a heterogeneous gamma fraction. The monoclonal protein was compatible with the clonal biology commonly observed in CAD, but by itself it neither established primary CAD nor excluded an overt B-cell lymphoproliferative neoplasm. Lymphoma or another marrow-based neoplasm could neither be excluded nor classified because no bone marrow examination or flow cytometry was performed. Specific testing for Mycoplasma pneumoniae or Epstein-Barr virus and a systematic autoimmune evaluation were not documented. The presentation was therefore classified as probable CAD associated with an unclassified IgM-kappa clonal process, while an occult lymphoproliferative neoplasm or another secondary trigger remained possible. Selected laboratory and immunohematologic findings are summarized in Table 2.

**Table 2:** Selected laboratory and immunohematologic findings Only reference intervals visible in the supplied laboratory records are reproduced; no generic intervals were substituted. Haptoglobin and peripheral-smear findings were unavailable. Indirect bilirubin was calculated from the documented total and direct values. CAD: cold agglutinin disease; DAT: direct antiglobulin test; IgM: immunoglobulin M; TIBC: total iron-binding capacity; TSAT: transferrin saturation; UIBC: unsaturated iron-binding capacity.

Test	Patient result	Institutional reference interval	Interpretation
Hemoglobin	5.1 g/dL on Day 0; 9.3 g/dL on Day 14	12.0-16.0 g/dL	Profound anemia with improvement after transfusion and supportive care
Lactate dehydrogenase	401 U/L	Not supplied	Supported hemolysis in the clinical context
Reticulocytes	4.78%	Not supplied	Reticulocytosis supported an erythropoietic response
Bilirubin	Total 1.10 mg/dL; direct 0.54 mg/dL; calculated indirect 0.56 mg/dL	Not supplied	Indirect fraction calculated as total minus direct
Iron profile	Iron 30 µg/dL; TIBC 341 µg/dL; UIBC 311 µg/dL; ferritin 41.7 ng/mL; calculated TSAT 8.8%	Not supplied; TSAT calculated from iron/TIBC	Marked iron restriction; supported concomitant iron deficiency
Direct antiglobulin test	Positive; C3d and IgM reactivity documented	Qualitative test	Supported cold-antibody-mediated hemolysis but was not specific for CAD
Serum cold-reactive antibodies	Detected	Qualitative finding	Titer and thermal amplitude were unavailable
Serum free kappa light chains	26.47 mg/L	3.3-19.4 mg/L	Mildly increased
Serum-free lambda light chains	31.80 mg/L	5.7-26.3 mg/L	Mildly increased
Beta-2 microglobulin	3.48 mg/L	0.80-2.34 mg/L	Increased; nonspecific in the setting of reduced renal function
Total serum protein	5.83 g/dL	6.4-8.3 g/dL	Decreased
Serum albumin	3.00 g/dL	4.02-4.76 g/dL	Decreased
Gamma globulin	0.91 g/dL (15.6%)	0.80-1.35 g/dL (11.1%-18.8%)	Within the reported interval; heterogeneous fraction prompted immunofixation
Serum immunofixation	Monoclonal IgM-kappa protein	Qualitative test	Supported an IgM-related clonal process requiring hematologic characterization

After probable CAD and the relevance of cold exposure were discussed, the patient and family reported that she had long avoided cold temperatures and kept her home unusually warm. She described severe pain after drinking or eating cold products and limb pain during cold weather, particularly in the preceding months. These symptoms had not been volunteered during the initial bleeding-focused evaluation. Cold avoidance was advised. The record did not document whether compatibility testing was performed at 37°C or whether blood warmers, warmed fluids, or active warming measures were used.

With transfusion support, diuresis, and other supportive treatment, dyspnea and edema improved and hemoglobin reached 9.3 g/dL by Day 14. Her mental and functional status also improved; by discharge, she was alert and appropriately oriented to self and her surroundings and was able to ambulate with assistance or a walker. A presumed urinary tract infection was treated with oral fosfomycin. She was discharged on Day 15 with hematology referral. At approximately 12 weeks after discharge, she was reported clinically stable under primary-care and family supervision. No follow-up hemoglobin or hemolysis markers were available. After the expected benefits and burdens were discussed, the patient, supported by her family, chose not to pursue invasive hematologic evaluation. The decision reflected her advanced age, frailty, comorbidities, and preference to avoid burdensome procedures when the results were unlikely to change the planned conservative management.

## Discussion

This case illustrates how profound cold agglutinin-mediated hemolysis may be obscured by competing explanations in a very old patient. Anticoagulant use, intermittent epistaxis, major structural heart disease, and fluid overload reasonably directed the initial evaluation toward bleeding and cardiac decompensation. The finalized blood-group confirmation result returned from the regional blood center reportedly noted cold-reactive antibodies by the second day and redirected the work-up toward immune hemolysis. This result was an early clue, not a diagnosis by itself. No overt bleeding was identified, and the convergence of reticulocytosis, elevated lactate dehydrogenase, a C3d-reactive DAT, serum cold-reactive antibodies, an IgM-kappa monoclonal protein, and chronic cold-dependent symptoms favored a pathogenic cold-antibody process over incidental cold reactivity. DAT positivity alone is not specific for CAD, and published diagnostic frameworks include a cold agglutinin titer of at least 1:64 at 4°C [2]. Because no titer was available and gastrointestinal bleeding was not completely excluded, probable CAD is more accurate than an unqualified assertion that every formal criterion was met.

Once cold-reactive antibodies had been identified, the differential diagnosis shifted to the cause and classification of cold-antibody-mediated hemolysis. CAD commonly reflects a characteristic IgM-producing marrow clone without overt malignancy, whereas secondary cold agglutinin syndrome accompanies an identified infection, systemic autoimmune disease, or overt malignancy [2,5]. Thus, an IgM-kappa monoclonal protein alone does not establish secondary disease. In this case, cross-sectional imaging did not identify definite lymphadenopathy or hepatosplenomegaly, and limited viral screening was nonreactive. However, specific testing for Mycoplasma pneumoniae or Epstein-Barr virus and systematic autoimmune testing were not documented. These findings did not exclude a marrow-limited clonal disorder, lymphoma, another neoplasm, or an untested infectious or autoimmune trigger. The absence of an on-site hematology department constrained inpatient classification; outpatient hematology evaluation was recommended, but invasive testing was subsequently declined. Accordingly, the most defensible classification was probable CAD associated with an unclassified IgM-kappa clonal process rather than confirmed primary CAD or confirmed secondary cold agglutinin syndrome.

The clinical pattern was concordant with classical complement-mediated CAD biology. IgM binding in cooler peripheral vessels activates the classical complement pathway; after rewarming, IgM generally dissociates, whereas C3 fragments remain on erythrocytes and promote predominantly hepatic extravascular clearance [2,3]. Thermal amplitude is clinically important because antibodies that react at temperatures reached in ordinary peripheral circulation may cause symptoms without extreme cold exposure [2]. The history of pain after cold food or beverages and cold-triggered limb pain was therefore not incidental. Its late recognition also shows why focused questions about temperature-related symptoms can be valuable when patients or families have normalized long-standing avoidance behaviors. Cold agglutination may also artifactually lower automated red-cell counts and increase derived indices such as mean corpuscular volume if samples are not adequately warmed [2]. Because specimen-handling conditions were unavailable, those indices should be interpreted cautiously.

The IgM-kappa monoclonal protein was consistent with the clonal biology commonly observed in CAD, but it did not by itself distinguish the characteristic CAD-associated marrow clone from an overt lymphoproliferative neoplasm. In the Norwegian population-based cohort, a monoclonal band was found in 94% of patients; among characterized proteins, 90% were IgM and 94% were kappa restricted [1]. Later work supports a distinct CAD-associated clonal B-cell disorder rather than classic lymphoplasmacytic lymphoma in many patients [2,4]. In this patient, however, the exact clonal entity could not be assigned without marrow morphology, flow cytometry, quantitative monoclonal-protein measurement, and a complete immunoglobulin assessment. The parallel mild elevations of free kappa and lambda light chains and beta-2 microglobulin were nonspecific, particularly with reduced renal function, and were not used to establish a diagnosis of monoclonal gammopathy of undetermined significance.

Hemoglobin of 5.1 g/dL lies within the severe range reported in CAD cohorts, and red-cell transfusion is common over the disease course [1,5]. In this patient, profound anemia likely reduced cardiopulmonary reserve in the setting of ischemic, valvular, and right-heart disease. Nevertheless, the data do not prove that anemia alone caused the heart failure episode. The transfusion event occurred in the setting of worsening peripheral edema, elevated N-terminal pro-B-type natriuretic peptide, major structural heart disease, and small pre-existing pleural effusions. Dyspnea developed during the second unit, the control radiograph showed increased pulmonary congestion and pleural fluid, and the patient later improved after intensified diuresis. This pattern favored an important circulatory or volume-overload component. However, no formal transfusion-reaction investigation, immediate post-event hemolysis panel, paired natriuretic-peptide measurement, fluid-balance record, or exact infusion rate was available. TACO therefore could not be formally classified, and an acute hemolytic or other transfusion reaction could not be definitively excluded. The different radiographic techniques further limit direct interval comparison.

Supportive management remains essential during acute CAD-related anemia. Expert reviews recommend keeping the patient and infusion extremity warm, performing compatibility testing at 37°C, and considering an in-line blood warmer when transfusion is required [2]. The available record did not establish which of these precautions were used. In this admission, later transfusions were completed under diuretic cover, and the patient improved with transfusion, diuresis, and cold avoidance. The available ferritin was 41.7 ng/mL and calculated transferrin saturation was 8.8%, providing a biochemical rationale for iron replacement. The pattern supported concomitant iron deficiency with marked iron restriction, although absolute depletion and inflammation-related functional restriction could not be separated. Because transfusions, diuresis, and iron were administered during the same admission, the specific contribution of iron to hemoglobin improvement cannot be determined.

Disease-directed treatment should be individualized. Corticosteroids are generally ineffective in typical CAD and are not recommended as routine therapy [2]. Prospective uncontrolled studies reported responses in approximately half of patients receiving rituximab alone, while fludarabine-rituximab and bendamustine-rituximab produced higher response rates but carried clinically important hematologic toxicity; cross-trial comparisons should be interpreted cautiously [6-8]. The C1s inhibitor sutimlimab rapidly reduced hemolysis and improved anemia in the single-group CARDINAL trial and in the randomized, placebo-controlled CADENZA trial [9,10]. Complement inhibition acts rapidly but does not eradicate the underlying B-cell clone. For this patient, clinical stabilization, improved mental and functional status, frailty, major comorbidity, and the patient-centered preference to avoid burdensome investigation when its results were unlikely to change conservative management supported follow-up rather than immediate clone-directed or complement-directed treatment.

This report has important limitations. The cold agglutinin titer, thermal amplitude, original detailed DAT report, haptoglobin, peripheral smear, complement concentrations, and specimen-handling conditions were unavailable. The full original blood-group report was unavailable for review, and its timing was reconstructed from the clinical team's chronology. Bone marrow evaluation and flow cytometry were not performed, targeted testing for selected infectious triggers and a systematic autoimmune evaluation were not documented, the gastrointestinal evaluation was incomplete, the available iron studies could not fully distinguish absolute iron depletion from inflammation-related functional restriction, and the exact infused portion and rate of the interrupted red-cell unit were not recorded. No formal transfusion-reaction investigation or immediate post-event hemolysis panel was available. Follow-up was clinical and did not include repeat hematologic results. These gaps limit formal classification but do not negate the convergent evidence for probable CAD associated with an unclassified IgM-kappa clonal process: hemolysis, C3d-reactive DAT, serum cold-reactive antibodies, an IgM-kappa monoclonal protein, and a strongly supportive cold-dependent history.

## Conclusions

Probable cold agglutinin disease should be considered in older patients with otherwise unexplained profound anemia and cardiopulmonary decompensation, particularly when a complement-reactive direct antiglobulin test accompanies evidence of hemolysis. Directed questions about pain after cold food or beverages, cold-triggered limb symptoms, and habitual avoidance of cool environments may reveal clues that were not initially volunteered.

When cold agglutinin titer, thermal amplitude, marrow evaluation, or other key tests are unavailable, the diagnostic wording should reflect that uncertainty. In this patient, occult gastrointestinal blood loss was not fully excluded, and the IgM-kappa monoclonal protein supported a possible CAD-associated clonal process whose exact nature remained unresolved without marrow evaluation. In frail patients with major cardiac comorbidity, management also requires a careful balance between transfusion support, volume status, warming precautions, the expected benefits and risks of disease-directed therapy, and the patient's goals of care.
